# Genome-Wide Survey Reveals Transcriptional Differences Underlying the Contrasting Trichome Phenotypes of Two Sister Desert Poplars

**DOI:** 10.3390/genes7120111

**Published:** 2016-12-01

**Authors:** Jianchao Ma, Xiaodong He, Xiaotao Bai, Zhimin Niu, Bingbing Duan, Ningning Chen, Xuemin Shao, Dongshi Wan

**Affiliations:** State Key Laboratory of Grassland Agro-Ecosystem, School of Life Sciences, Lanzhou University, Lanzhou 730000, China; majch13@lzu.edu.cn (J.M.); 18794241141@163.com (X.H.); bxt15138691581@163.com (X.B.); niuzhm16@lzu.edu.cn (Z.N.); duanbb14@lzu.edu.cn (B.D.); Chennn15@lzu.edu.cn (N.C.); shaoxm15@lzu.edu.cn (X.S.)

**Keywords:** *P. euphratica*, *P. pruinosa*, trichome, C2H2, MYB, bHLH, differentially expressed genes

## Abstract

Trichomes, which are widely used as an important diagnostic characteristic in plant species delimitation, play important roles in plant defense and adaptation to adverse environments. In this study, we used two sister poplar species, *Populus pruinosa* and *Populus euphratica*—which have, respectively, dense and sparse trichomes—to examine the genetic differences associated with these contrasting phenotypes. The results showed that 42 and 45 genes could be identified as candidate genes related to trichomes in *P. pruinosa* and *P. euphratica*, respectively; most of these genes possessed high degrees of diversification in their coding sequences, but they were similar in intron/exon structure in the two species. We also found that most of the candidate trichome genes were expressed at higher levels in *P. pruinosa*, which has dense trichomes, than in *P. euphratica*, where there are few trichomes. Based on analyses of transcriptional profiles, a total of 195 genes, including many transcription factors, were found to show distinct differences in expression. The results of gene function annotation suggested that the genes identified as having contrasting levels of expression level are mainly associated with trichome elongation, ATPase activity, and hormone transduction. Changes in the expression of these and other related genes with high sequence diversification may have contributed to the contrast in the pattern of trichome phenotypes between the two species.

## 1. Introduction

The trichome, an important phenotypic characteristic found widely in unrelated plants, is used in the delimitation and identification of plant species [1,2]. Trichomes are usually located on aerial tissues such as leaves, stems, petioles, and seed coats, where they can protect plants from adverse conditions, including insect and pathogen attack as well as temperature extremes and excessive light [3,4]. Trichomes are enlarged and modified epidermal cells that grow perpendicular to the plant surface; they are produced by specialized processes of cellular differentiation, development, and repatterning [3,5]. It has been suggested that the formation of trichomes associated with the processes of plant development and adaptation of plants to various environments is regulated by complex networks in which both endogenous and environmental signals are involved [6,7,8].

Multiple lines of evidence from the model plant *Arabidopsis* suggest that three major groups of transcription factors (TFs) are involved in regulating the development of trichomes and their spatial and temporal distribution. Firstly, several genes of the basic helix-loop-helix (bHLH) family, such as *GLABRA3* (GL3) and *ENHANCER OF GLABRA3* (EGL3), are involved in trichome and root-hair differentiation [9,10]. Secondly, it has been suggested that the products of some members of the R3 family of MYB genes, *TRICHOMELESSs* (TCLs), *CAPRICE* (CPC), *TRYPTICHON* (TRY) and their enhancers *CPC1* and *CPC2* (ETC1 and ETC2), which belong to R3 repeatedly, negatively regulate trichome formation [11,12,13,14], while several R2R3 MYB gene products including MYB0, 23, 82, and 106 positively regulate trichome formation [15,16,17,18]. Thirdly, several genes of the C2H2 family, such as *ZINC FINGER PROTEIN*s (ZFPs) and *GLABROUS INFLORESCENCE STEMS* (GIS) regulate trichome initiation by integrating gibberellin (GA) and cytokinin signaling [19,20,21]. In addition to these genes, accumulating evidence suggests that phytohormones such as gibberellins (GAs), cytokinins (CKs), and jasmonic acids (JAs) participate in trichome development [7,22]. Furthermore, some functional genes including *TRANSPARENT TESTA GLABRA* (TTG1), *SPINDLY* (SPY), and *REPRESSOR OF ga1-3* (RGA) appear to be involved in trichome formation [23,24]. Some functional homologs of the transcription factors that regulate trichome formation have been identified in other plants. For example, in cotton, *GaHOX1* and *GaMYB25* have been shown to be functional homologs of GL2 [25] and MYB106 [26], respectively. Several MYB-like genes from *Mimulus guttatus* and peach have been found to regulate trichome formation [27,28]. The expression of a tomato R3 MYB gene resulted in glabrous phenotypes (phenotypes of no trichome) in *Arabidopsis* [6]. In *Populus*, *PtrTCL1-8*, a functional homolog of TCL1 (which belongs to the R3 MYB family) in *Arabidopsis* [29], and *PtrMYB168* (which belongs to the R2R3 MYB family), have recently been reported to regulate trichome formation [8]. These results suggest that the mechanisms of trichome formation in other plant species, such as poplar, may be similar to that in the model species *Arabidopsis*.

Two sister plant species with disparate trichome densities provide a good model system in which to uncover the genetic differences underlying trichome formation because such species are likely to have diverged recently, in response to selective pressures in contrasting habitats, from a recent common ancestor. In this study, we aimed to examine the genetics of differing patterns of trichome formation in two sister poplar species, *Populus euphratica* Oliv. and *P. pruinosa* Schrenk. Although both species occur in central Asia and their distributions overlap, *P. euphratica* prefers dry central deserts with deep underground water, while *P. pruinosa* is distributed in deserts near to current or ancient rivers where the atmosphere is wet [30,31]. These two species diverged from each other recently, between 0.5 and 2 million years ago [32], but they exhibit different ecological adaptations to their differentiated habitats. For example, *P. euphratica* has a higher tolerance of drought stress than *P. pruinosa*, while *P. pruinosa* shows higher salt tolerance than *P. euphratica* [32]. In addition, the two species show well-differentiated morphology. For example, leaves of seedlings in *P. euphratica* are lanceolate with few trichomes, while those of *P. pruinosa* are ovate or kidney-shaped with a high density of trichomes. Previous studies have shown that these two sister poplars have evolved different genetic strategies for salt and drought resistance [32,33,34,35,36]. However, it is not known whether genetic variations also underlie the differences in trichome phenotype in these two species. Here, we firstly examined changes in numbers of candidate trichome genes relative to the numbers of homologs identified in *Arabidopsis*. Next we investigated changes in expression of these candidate trichome genes. Finally, we used transcriptome analyses to examine other differentially expressed genes that may be involved in the contrast in the pattern of trichome phenotypes between these two poplar species.

## 2. Materials and Methods

### 2.1. Plant Materials and Growth Conditions

One-year-old seedlings (both planted in March 2014) of *P. euphratica* and *P. pruinosa* were grown in a greenhouse at 25 °C with cycles of 16 h light/8 h darkness (100 µmol·m^−2^·s^−1^), which is favorable to both species. Only healthy seedlings were used in this experiment. The youngest leaves of the seedlings, which were used for the extraction of total RNA, were collected at a similar growth stage for the two poplar species; three seedling individuals of each species were selected as biological replicates.

### 2.2. Total RNA Extraction and Sequencing

Total RNA for transcriptome sequencing was extracted from leaves of the two species at the trichome production stage using a cetyltrimethyl ammonium bromide (CTAB) procedure [37], and the concentrations of RNA samples were quantified using a NanoDrop 1000 (Thermo Scientific Inc., Wilmington, DC, USA). cDNA libraries from these samples were constructed and sequenced at Biomarker Technologies (Beijing, China) following standardized procedures and monitored using a standard quality control system. In brief, RNA with poly(A) tails was purified from total RNA using oligonucleotide (dT) magnetic beads and fragmented into short sequences, which were used for template cDNA synthesis. After end repair, adapter ligation, and PCR amplification, paired-end cDNA libraries were constructed and then sequenced using an Illumina HiSeq 2500 sequencing platform (Illumina Inc., San Diego, CA, USA) with a 150 bp read length. Image output data from the sequencer were transformed by base-calling into raw sequence data, which were stored in the FastQ format. The sequencing data was then deposited in the NCBI Sequence Read Archive database (SRA; http://www.ncbi.nlm.nih.gov/sra) with accession number SRP070798.

### 2.3. Identification of Genes Involved in Trichome Formation

Candidate genes related to trichome formation in *P. euphratica* [34], *P. pruinosa* (Supplementary Material, Table S1) and *P. trichocarpa* [38] were identified using the BLASTP program (E values < 10^−50^) [39] and HMMER software [40] with similarity over 90%. Trichome-formation genes from *Arabidopsis* downloaded from the *Arabidopsis* genome website TAIR 9.0 (http://www.Arabidopsis.org/index.jsp) were used as query sequences. Phylogenetic trees were constructed using the genes identified as being involved in trichome formation in poplars and *Arabidopsis* in order to determine the relationships between these genes. Candidate genes in *P. trichocarpa*, which have sparse trichome density, were used as a reference to explore gene expansion and lose in poplar.

### 2.4. Phylogenetic Analyses and Structure of Candidate Genes

Candidate genes from the four species were aligned by MUSCLE [41] and then adjusted manually before construction of phylogenetic trees. The best-fitting evolutionary models were predicted using the program ProtTest 3.0 [42]. Gene trees for the different transcription factors were estimated, using a maximum likelihood (ML) approach with the best-fitting models and 1000 replicates, by RAxML software [43] and constructed using MEGA 6 [44]. Chromosomal distribution analysis was performed using the method described in Ma et al. [45].

### 2.5. dN/dS Calculation and Inference of Divergence Time

Pairwise alignments of the nucleotide sequences of homologous genes were performed using the Probabilistic Alignment Kit (PRANK) software package [46]. The nonsynonymous substitution (dN or Ka) and synonymous substitution (dS or Ks) values for homologous genes were estimated by the YN00 program in Phylogenetic Analysis Using Maximum Likelihood (PAML) [47]. The synonymous substitution rates (Ks) for homologous genes would be expected to be similar over time and could be used as a proxy for time in order to estimate the dates at which segmental duplication events occurred. The Ks value was calculated for each of the gene pairs and used to calculate the approximate date of each duplication event (T = Ks/2λ), assuming a clocklike rate (λ) of synonymous substitution of 9.1 × 10^−9^ substitutions/synonymous site/year for *Populus* [48].

### 2.6. Gene Expression Data

Raw reads were cleaned firstly by removing exact duplicates obtained from both sequencing directions and secondly by removing adapter sequences and reads for which unknown base calls (N) represented more than 5% of all bases; low complexity reads; and reads with high proportions of low-quality bases (>45% of the bases with a quality score ≤7).

To determine levels of gene expression, Bowtie 2 [49] was used to align RNA-Seq reads to each poplar genome. Transcript abundances were calculated using eXpress [50], which outputs read counts and the number of fragments per kilobase of exon per million fragments mapped (FPKM) [51], and the average FPKM values were calculated from three biological replicates. The FPKM values for genes related to trichome production were log2-transformed and used for heat map generation with the pheatmap package in R.

### 2.7. Identification and Functional Analysis of Differentially Expressed Genes (DEGs)

To identify DEGs between seedling leaves of the two poplar species, which have significantly different degrees of trichome cover, fold changes in the levels of expression of homologous genes were computed. DEGs between the two species, on the basis of three biological replicates of each, were identified using the Empirical Analysis of Digital Gene Expression data package in R (edgeR) ver. 2.6.12 (www.cran.rproject.org). In carrying out pairwise comparisons with edgeR, estimateGLMCommonDisp and exactTest settings were applied, and the *p*-values obtained were adjusted using the false discovery rate (FDR) by function *p*.adjust in R. An absolute fold change of >2 and an FDR significance score of <0.001 were used as thresholds to identify significant differences in gene expression. Each DEG was annotated, initially according to the top *Arabidopsis* hit and then by Gene Ontology (GO) enrichment analysis, which was performed using GO Slim (http://www.geneontology.org/page/go-slim-and-subset-guide), to assign it to the one of the three principal GO categories: molecular function, cellular component, and biological process.

### 2.8. Validation of Genes Related to Trichome Formation with Quantitative Real-Time PCR (qRT-PCR)

To validate the reliability of the RNA-Seq analyses, eight candidate gene pairs related to trichome formation were selected for quantitative RT-PCR (qRT-PCR) tests. A sample of 0.5 μg of DNase I-treated total RNA was transcribed into single-stranded cDNA using a PrimeScript 1st Strand cDNA Synthesis Kit (TaKaRa, Dalian, China). The cDNA templates were then diluted 20-fold before use. The quantitative reactions were performed on a CFX96 Real-Time PCR Detection System (Bio-Rad, Singapore) using SYBR Premix Ex Taq™ (TaKaRa, Dalian, China) with the following temperature program: 30 s at 95 °C, followed by 40 cycles of 95 °C for 15 s, 60 °C for 30 s, and finally 72 °C for 20 s. All gene-specific primers, which were designed using the PRIMER 5 software package [52], are listed in Table S2 of Supplementary Material. Three biological replicates were used for each gene. Relative expression levels were normalized to the level of expression of the internal reference gene β-actin and calculated using the 2^−∆∆Ct^ method [53].

## 3. Results and Discussion

### 3.1. Trichome Differences in Two Sister Desert Poplars

**Figure 1:**
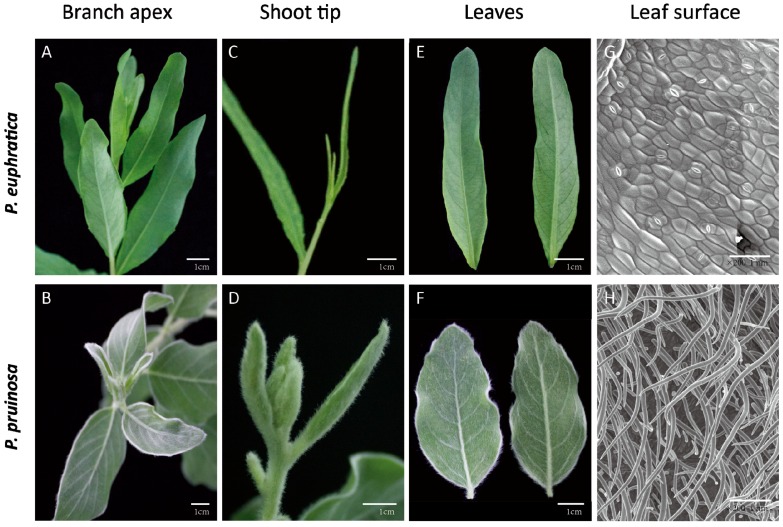
Trichome phenotypes of *P. pruinosa* and *P. euphratica*. An absence of trichomes was observed on *P. euphratica* branch apices, shoot tips and leaves (**A**, **C**, and **E**); while dense trichomes were seen on *P. pruinosa* (**B**, **D**, and **F**); Scanning electron microscopy (**G**, **H**) images of leaves from *P. pruinosa* and *P. euphratica*, respectively. Trichomes with a glabrous phenotype were observed in *P. euphratica*.

The trichome is an important diagnostic character used in plant identification and plant taxonomy, and is involved in deterring herbivore attack and in adaptation to adverse environments [3,4,54]. Unlike *A. thaliana*, poplar trichomes never undergo branching. In this study, we investigated the distribution of trichomes on the seedling leaves of two desert poplar species. As shown in Figure 1, a high density of trichomes was observed on the seedling leaves and branches of *P. pruinosa*, whereas there were few trichomes on those of *P. euphratica* (Figure 1A–F). Similar results were obtained using scanning electron microscopy (Figure 1G,H). The markedly different patterns of trichomes on their seedling leaves suggested that the two poplars may be exposed to different threats during the seedling development stages, so that in order to thrive in diverse environments, the plants have evolved different adaptive traits. For example, high-density trichomes on seedling leaves may assist *P. pruinosa* in defending itself against insect attack and excessive evapotranspiration in desert environments, while in *P. euphratica* seedlings, lanceolate leaves may be more suited to preventing insect attack and reducing evapotranspiration. Differences in leaf shape and trichome pattern may therefore work together to assist *P. euphratica* and *P. pruinosa* in adapting to desert environments [32].

### 3.2. Identification of Genes Related to Trichome Formation

**Figure 2:**
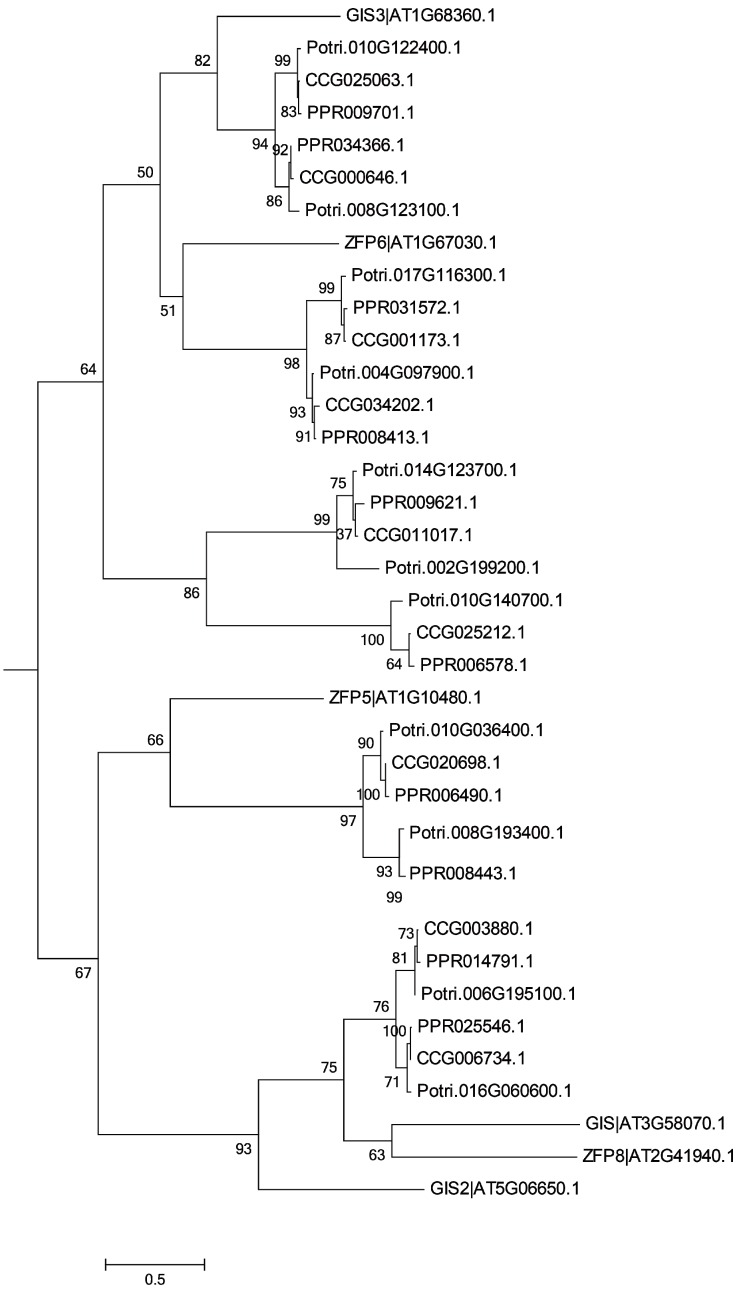
Phylogenetic relationships of C2H2 transcription factors (TFs) from *Arabidopsis*, *P. trichocarpa*, *P. pruinosa*, and *P. euphratica*. The phylogenetic tree was built using the maximum likelihood method implemented in RAxML. Homology of genes between *Arabidopsis* and the three poplars was confirmed using the tree.

**Figure 3:**
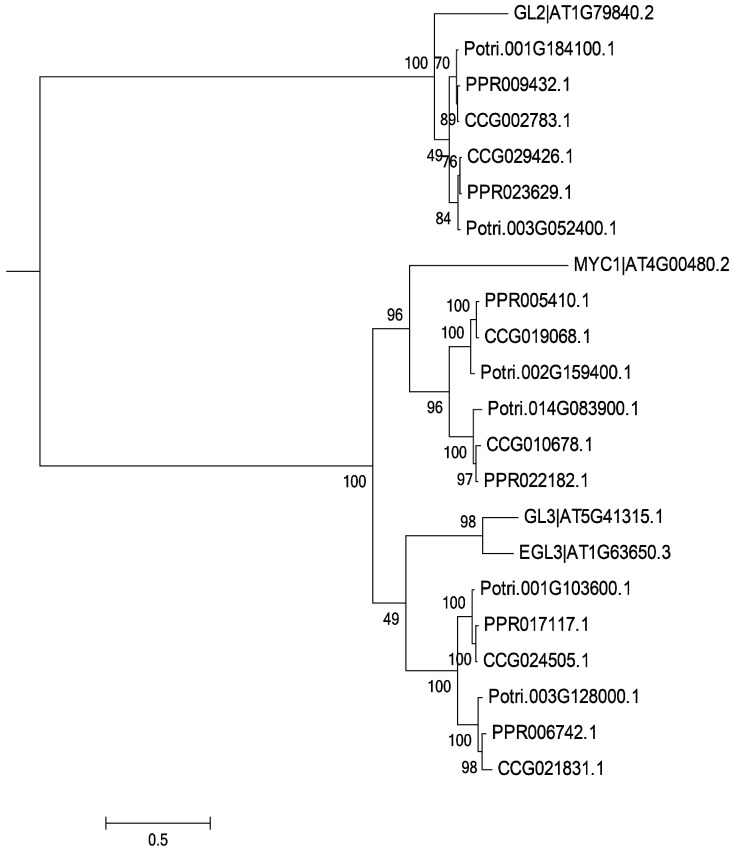
Phylogenetic relationships of basic helix-loop-helix (bHLH) TFs (except for GL2) from *Arabidopsis*, *P. trichocarpa*, *P. pruinosa*, and *P. euphratica*. The phylogenetic tree was built using the maximum likelihood method implemented in RAxML. Homology of genes between *Arabidopsis* and the three poplars was confirmed using the tree.

**Figure 4:**
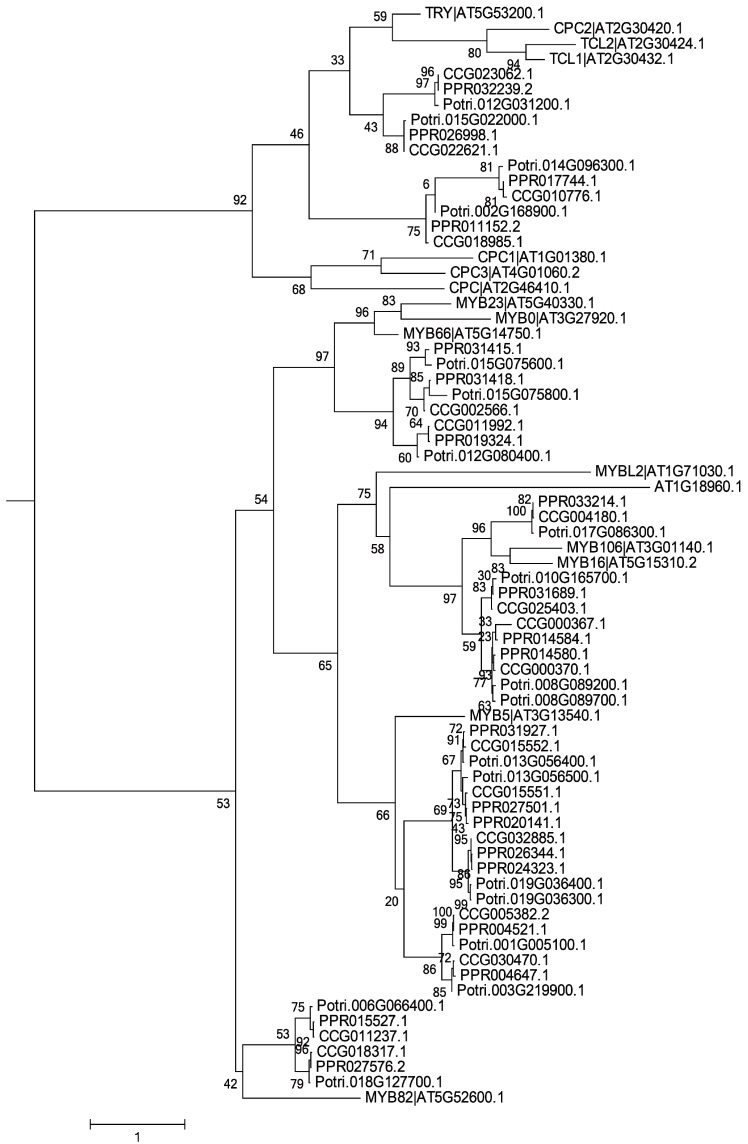
Phylogenetic relationships of MYB TFs from *Arabidopsis*, *P. trichocarpa*, *P. pruinosa*, and *P. euphratica*. The phylogenetic tree was built using the maximum likelihood method implemented in RAxML. Homology of genes between *Arabidopsis* and the three poplars was confirmed using the tree.

Trichome-formation genes have been well characterized in *Arabidopsis* [7,9,10,11,12,13,14,15,16,17,18,19,20,21]**,** and homologs of these genes have been shown to encode products with similar functions in other species [27,28,29]. To find potential regulators of trichome formation in the two desert poplars, we surveyed the genomes of *P. euphratica* [34] and *P. pruinosa* (Supplementary Material, Table S1). Forty-two and 45 genes potentially related to trichome formation, which were homologous to *Arabidopsis* genes (with sequence similarity >90%), were characterized in *P. euphratica* and *P. pruinosa*, respectively (Supplementary Material, Table S3). Phylogenetic analysis showed that 70% of these genes could be assigned to one of three families of transcription factors—the C2H2, bHLH, and MYB transcription factors (Figure 2, Figure 3 and Figure 4)—suggesting that most gene families related to trichome formation present in *Arabidopsis* are also found in the two desert poplars, but that they are greatly expanded in poplars. For instance, there are two members of the *AtMYB5* group in *Arabidopsis*, but they have up to five homologs in the poplars. By examining the chromosomal distribution of the genes, we found that most of the expanded gene families had arisen from whole genome duplication (WGD) events [38], though several gene pairs which were the homologs of *AtMYB5*, *AtMYB106*/*16*, and *AtZFP5* had experienced tandem duplication events. The structures of homologs in *P. euphratica* and *P. pruinosa* showed that most candidate genes shared similar exon–intron structures between the two poplars. In examining their chromosomal distribution, no clustering of gene pairs was found, indicating that these genes were not acquired recently (since this would have allowed insufficient time for dispersal), but rather that it is variation in existing genes that delivers the different trichome phenotypes in poplars.

**Figure 5:**
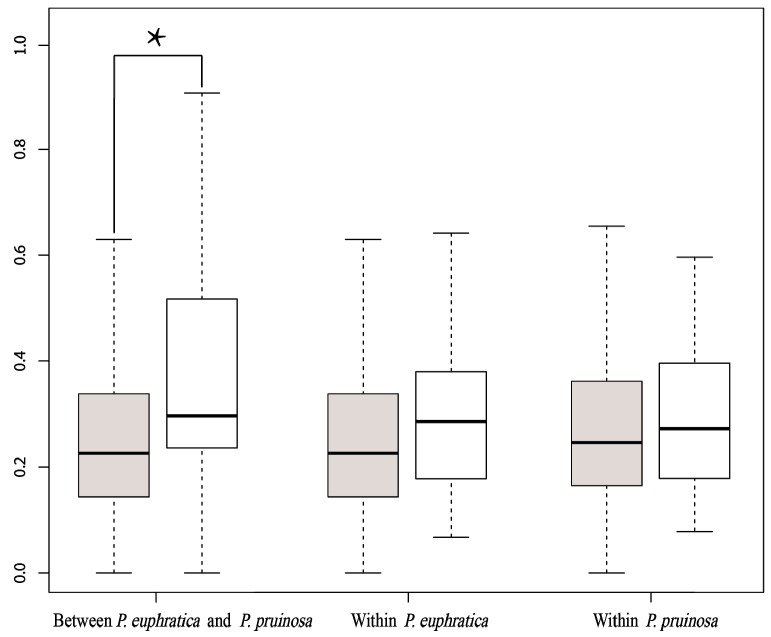
Box plot of nonsynonymous substitution/synonymous substitution (dN/dS) ratios for gene pairs either involved or not involved in trichome formation. Boxes shaded grey show the dN/dS ratios of gene pairs not related to trichome formation. The remainder represent the dN/dS ratios of gene pairs involved in trichome formation. The trichome formation-associated gene pairs have higher dN/dS ratios. Asterisks indicate statistically significant differences.

We calculated the rates of nonsynonymous (dN) and synonymous substitution (dS) per site between homologous genes. The average dN/dS ratio of the homologous genes related to trichome formation was 0.38, which is much higher than the average dN/dS ratio (0.26) of other orthologous genes detected in the *P. euphratica* and *P. pruinosa* genomes (Table S4 (Supplementary Material) and Figure 5). The results were supported by a Kolmogorov–Smirnov test (*p*-value = 0.027). Nonsynonymous single nucleotide polymorphisms introduce amino acid changes into the corresponding proteins, and this may lead to changes in gene function [55]. Our estimates indicated that genes related to trichome formation may have undergone rapid divergence between these two sister desert poplars. We also estimated evolutionary rates for these homologous genes using Ks as the proxy (Supplementary Material, Table S5). The results indicated that most trichome candidate gene pairs began to differentiate about one million years ago (Mya), which is largely consistent with previous estimates of the divergence time (about 0.5–2 Mya) of these two species [32,35,36], suggesting that most trichome candidate genes began to diversify following the divergence of the two species and that some mutant sites became fixed in the genome, which could have contributed to adaptations to diverse environments, such as the production of dense trichomes in seedling leaves of *P. pruinosa*.

### 3.3. Expression of Candidate Trichome-Formation Genes

**Figure 6:**
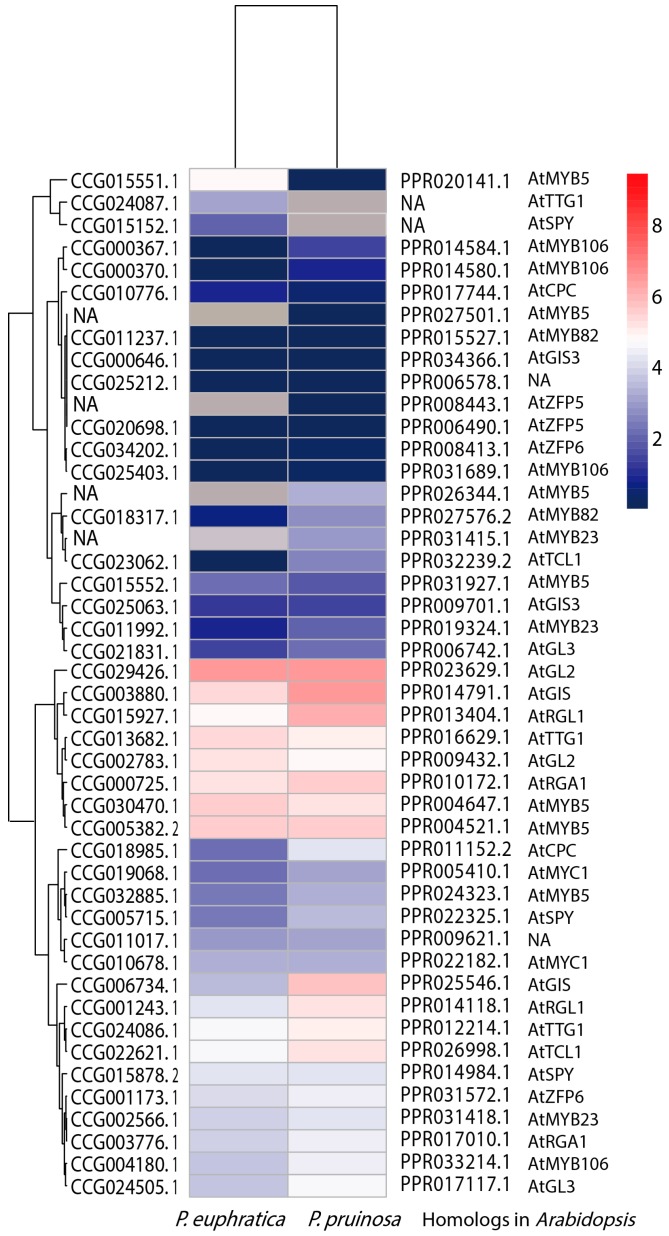
Expression of trichome-formation genes compared between *P. pruinosa* and *P. euphratica*. Heatmap of the expression of trichome formation genes in *P. pruinosa* and *P. euphratica*. The Illumina RNA-Seq data were analyzed, the fragments per kilobase of exon per million fragments mapped (FPKM) values were log2-transformed, and a heatmap was generated. NA indicates that no homologous genes exist.

To determine whether the levels of expression of our candidate genes in the two sister poplars were associated with variation in trichome formation, an RNA-Seq approach was used to compare the seedling leaves of the two species. A total of 28 G (gigabyte) reads were sequenced (Supplementary Material, Table S6). The expression levels of all candidate genes were screened (Table S3 (Supplementary Material) and Figure 6). According to the FPKM values and heatmap clustering, most candidate genes in *P. euphratica* showed relatively low levels of expression compared with their homologs in *P. pruinosa*, though there were exceptions such as the gene pairs *CCG015551.1* (*PeuMYB5)* and *PPR020141.1* (*PprMYB5*) which may negatively regulate trichome formation [8,56]. We also investigated the levels of expression of candidate genes involved in signaling pathways regulating trichome cell differentiation in *Arabidopsis*, as reported previously [7]. Again, most genes showed relatively low levels of expression in *P. euphratica*, apart from the homologs of *CCG025063.1* (*PeuGIS3*) and *PPR009701.1* (*PprGIS3*), *CCG029426.1* (*PeuGL2*) and *PPR023629.1* (*PprGL2*), and *CCG024086.1* (*PeuTTG1*) and *PPR012214.1* (*PprTTG1*), which showed similar expression levels in the two species. Further analyses revealed that genes whose products act as negative regulators [7] also displayed relative low expression levels in *P. euphratica*. These findings indicated that the absence or presence of trichomes in these two poplars is probably related mainly to the expression levels of positive regulators of trichome-formation genes, and the regulation of trichome formation is a complicated process.

In general, the expression of genes is regulated through their upstream regulatory regions. We therefore analyzed the promoter regions (1500 bp upstream) of candidate genes using PlantCARE [57] to explore whether any difference in these regulatory regions could result in the observed differences in gene expression. A total of 15 cis-acting regulatory elements (CAREs) identified from 87 candidate genes are listed in Table S7 of Supplementary Material. Two to 5 CAREs were found per gene. When we compared the two poplar species, we found that most pairs of orthologs showed differences in the numbers and types of elements such as gibberellin response elements (GAREs), abscisic acid response elements (ARE), MYB-binding sites (MBS), and pyrimidine box (P-box) and W box elements. For example, *PPR023629.1* (*PprGL2*) has GAREs which are absent from *CCG029426.1* (*PeuGL2*). Since variations in the numbers and types of such elements could give rise to different levels of expression of orthologous genes, these results suggest that the orthologs may be regulated differently by the corresponding hormones or transcription factors.

Among these candidate genes, one MYB transcription factor, *PtaMYB186*, which was previously cloned from a poplar species, has been found to increase trichome density when overexpressed [16,29]. Other MYB transcription factors have also been reported to be involved in trichome formation in many other species, including cotton, *Mimulus guttatus*, and peach [27,28,58]. In our study, four homologs of these MYB transcription factors were recovered from the two poplar species. All of them showed much lower levels of expression in *P. euphratica* than in *P. pruinosa*. Of particular note, the FPKM values for the homologs of *MYB186*, *CCG000367.1* (*PeuMYB186*) and *PPR014584.1* (*PprMYB186*), were 0.55 and 2.74, respectively, indicating that the different expression of these genes may have made a major contribution to the development of the dense trichome phenotype in *P. pruinosa* seedling leaves.

**Figure 7:**
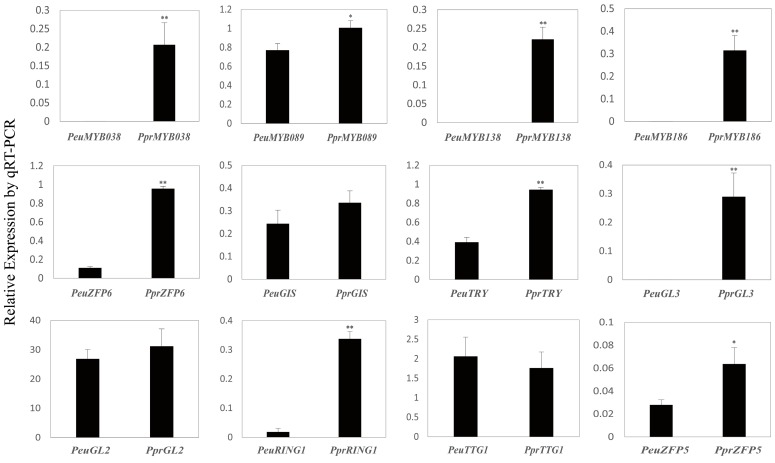
Verification of transcriptome data by quantitative real-time PCR (qRT-PCR). Eight gene pairs related to trichome formation were selected for qRT-PCR verification. All showed similar patterns of expression to those identified from transcriptome data. The vertical bars indicate standard deviations. Asterisks indicate statistically significant differences using Student’s *t*-test (**: *p* < 0.001; *: *p* < 0.05).

In order to confirm the levels of gene expression estimated from RNA-Seq data, a total of 12 candidate gene pairs from *P. euphratica* and *P. pruinosa* were selected for qRT-PCR analyses, including four pairs of homologs of *AtMYB106* and *AtMYB16*, two pairs of *ZFP* genes, *RING1*, *TRY*, and *GL3*, which were expressed significantly differently between the two desert poplars. We also examined three gene pairs, *GIS*, *GL2,* and *TTG1*, whose expression levels showed no obvious differences except *GL2*. *GL2* showed a higher expression in *P. pruinosa* in qRT-PCR but reversed in RNA-Seq results, indicating the expression of *GL2* is probably a time-point during development. Most qRT-PCR estimates were consistent with the RNA-Seq data for the same genes (Figure 7), suggesting that most of the candidate gene expression levels determined by RNA-Seq results are reliable.

### 3.4. Other Genes Identified among DEGs Potentially Related to Differences in Trichome Formation between the Two Species

**Figure 8:**
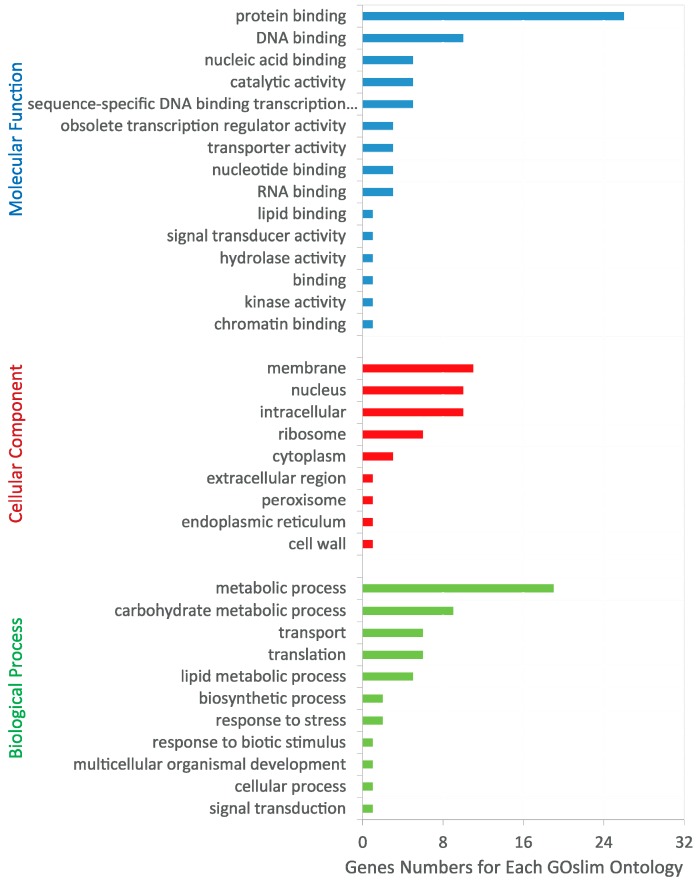
Gene Ontology (GO) Slim classification of DEGs between *P. pruinosa* and *P. euphratica* young leaves. DEGs were first annotated with GO terms and then classified by GO Slim analysis. The results are summarized for the three main GO categories: biological process, cellular component, and molecular function.

In total, we identified 195 differentially expressed genes (DEGs) that were characterized by having either higher or lower levels of transcript accumulation in seedling leaves of one of the two poplar species compared to the other (fold change >2 or <0.5, FDR <0.001) (Supplementary Material, Table S8). From the annotations associated with the homologous genes in *Arabidopsis*, we found that most of the genes identified in this way probably had functions related to cell wall biosynthesis, cell differentiation, trichome branching, hormone biosynthesis, photosynthesis, and responses to biotic and abiotic stresses [34,35,36,38] (Supplementary Material, Table S8). Of these, 30 DEGs related to trichome formation or trichome metabolic pathways are listed in Table 1. For instance, *PPR012085.1* (here named *PprRING1*), a homolog of *GhRING1*, which is expressed during cotton fiber development [59], consistent with qRT-PCR results, showed significantly higher levels of expression (log_2_FC = 12.09) in *P. pruinosa*, while *PPR012270.1*, another gene with increased expression in *P. pruinosa*, is a homolog of *AtPP2-A9*, which was previously reported to be involved in trichome production in *Arabidopsis* [16]. Furthermore, genes encoding some negative regulators were found to be downregulated in the seedling leaves of *P. pruinosa*; for example, *PprMYB5* (*PPR027501.1*), whose homolog *AtMYB5* has been reported to act as a negative regulator of trichome branching (Table 1) [56], was downregulated (log_2_FC = −5.64) in *P. pruinosa*. Of the DEGs, 152 could be annotated using WEGO [60] and GO Slim. In WEGO analysis, we only found “extracellular region” overrepresented in DEGs compared to the whole genome genes (Supplementary Material, Figure S1). In GO Slim analysis, we found that 54 of these genes appear to function in metabolic processes and the production of defense-related compounds (Figure 8), which suggests that most DEGs are involved in plant defense responses.

Some hormones, such as GA, cytokinins, JA, and SA, have been found to play important roles in trichome production or differentiation [22,61,62]. Among the DEGs, five genes were found to be involved in hormone transduction. One cytokinin signal transduction gene—*PPR033783.1*, a homolog of *AT5G49720*—was expressed differently in seedling leaves of the two poplars (Supplementary Material, Table S8). SA has a negative effect on trichome production and consistently reduces the effects of jasmonic acid [61]. In our study, one SA signal transduction gene—*CCG019378.1*, a homolog of *AT5G43940*—was expressed differently in the two species, showing a higher expression level in *P. euphratica* than in *P. pruinosa* (Supplementary Material, Table S8).

Trichome formation and elongation are energetically “costly” processes [63]. Interestingly, several DEGs (*PPR020233.1*, *PPR017561.1*, and *PPR00861.1*) were found to be involved in ATPase activity. These ATPase activity-related genes may contribute to *P. pruinosa* producing denser and longer trichomes on its leaves and stems, which could improve its adaptation to desert environments by increasing its resistance to, for example, drought stress, insect and pathogen attack, extremes of temperature, and/or excessive light. However, only about five DEGs were found to be involved in trichome formation; about 20 genes were associated with trichome elongation and ATPase activity. These results suggest that most trichome-related DEGs are involved in the processes of trichome elongation and ATPase activity. The DEGs *PPR020233.1*, *PPR017561.1*, and *PPR00861.1* may be important in trichome initiation in seedling leaves of *P. pruinosa*. Further research focusing on these issues will provide important resources for additional functional analyses and transgenic modification in order to develop more stress-tolerant poplars.

## 4. Conclusions

*P. pruinosa* and *P. euphratica* are two closely related desert poplars. The dense trichomes of *P. pruinosa* represent an important adaptive trait in desert environments. In this study, we have identified genes potentially involved in trichome formation in the two poplars through comparison with homologs in *Arabidopsis*, and examined their expression levels using RNA-Seq. We found that 80% of these candidate genes, which are expressed at higher levels in *P. pruinosa* than in *P. euphratica*, may be involved in cell wall biosynthesis, cell differentiation, trichome branching, hormone biosynthesis, photosynthesis, and responses to stresses, indicating the factors that contribute to the higher density of trichomes in *P. pruinosa*. DEGs related to ATPase activity and hormone transduction are likely to be involved in trichome formation and elongation in *P. pruinosa*. Our results shed new light on the genetic mechanisms underlying the contrasting trichome phenotypes of *P. pruinosa* and *P. euphratica*.

## Figures and Tables

**Table 1:** List of 30 differentially expressed genes (DEGs) between two poplar species.

*P. euphratica*	*P. pruinosa*	Fold Change (Log2)	Description	FDR
CCG018012.1	PPR012085.1	12.09	RING/U-box superfamily protein	1.86 × 10^−26^
CCG022412.1	PPR012270.1	11.58	phloem protein 2-A9	1.79 × 10^−6^
CCG008934.1	PPR034607.1	10.76	NAD(P)-linked oxidoreductase superfamily protein	1.80 × 10^−9^
CCG007202.1	PPR022993.1	10.62	cytochrome P450, family 76, subfamily C, polypeptide 1	2.93 × 10^−11^
CCG029108.1	PPR006882.1	9.67	NAD(P)-binding Rossmann-fold superfamily protein	3.68 × 10^−24^
CCG018958.1	PPR009435.1	7.10	Integrase-type DNA-binding superfamily protein	6.32 × 10^−4^
CCG015488.1	PPR011118.1	5.68	GDSL lipase 1	4.51 × 10^-18^
CCG018718.1	PPR024143.1	5.51	galacturonosyltransferase 4	1.74 × 10^-5^
CCG027592.1	PPR008974.1	5.01	kinase interacting family protein	1.11 × 10^-12^
CCG031474.1	PPR002601.2	4.63	peroxisomal 3-ketoacyl-CoA thiolase 3	1.53 × 10^−5^
CCG011904.1	PPR009473.1	4.54	photosystem I light harvesting complex gene 2	8.72 × 10^−7^
CCG009982.1	PPR018746.1	4.29	UDP-glucosyl transferase 85A3	7.02 × 10^−8^
CCG023470.1	PPR009451.1	3.99	acyl-CoA oxidase 2	2.53 × 10^−13^
CCG029588.1	PPR033783.1	3.60	glycosyl hydrolase 9A1	4.76 × 10^−5^
CCG010637.1	PPR021244.1	2.86	cyclin-dependent kinase D1;1	2.52 × 10^−4^
CCG025296.1	PPR010369.1	−3.16	heat stable protein 1	1.86 × 10^−5^
CCG025295.1	PPR010368.1	−3.72	heat stable protein 1	1.76 × 10^−5^
CCG033444.1	PPR004735.1	−4.48	wall-associated kinase 2	1.01 × 10^−6^
CCG004931.2	PPR029777.1	−4.51	basic chitinase	1.99 × 10^−6^
CCG023084.1	PPR032261.1	−5.46	chitinase A	7.02 × 10^−6^
CCG015551.1	PPR027501.1	−5.64	myb domain protein 5	1.31 × 10^−6^
CCG020573.1	PPR005272.1	−5.73	membrane bound O-acyl transferase family protein	4.47 × 10^−34^
CCG025292.1	PPR010363.1	−6.09	heat stable protein 1	2.35 × 10^-7^
CCG024468.1	PPR019009.1	−6.87	osmotin 34	1.07 × 10^−4^
CCG025293.1	PPR010365.1	−7.26	heat stable protein 1	1.84 × 10^−16^
CCG028100.1	PPR009517.1	−8.56	Matrixin family protein	1.534 × 10^−13^
CCG025849.2	PPR014894.1	−9.36	glucuronidase 3	2.03 × 10^−4^
CCG009602.1	PPR019463.1	−9.37	FAD/NAD(P)-binding oxidoreductase family protein	7.89 × 10^−7^
CCG006080.1	PPR019259.1	−10.16	amino acid transporter 1	1.95 × 10^−8^
CCG021502.1	PPR031281.1	−11.87	Esterase/lipase/thioesterase family protein	4.60 × 10^−7^

## Supplementary Materials

The following are available online at www.mdpi.com/2073-4425/7/12/111/s1, Figure S1: Go annotation of DEGs and whole genome genes by WEGO, Table S1: Sequences of *P. pruinosa*, Table S2: Primers used for real-time quantitative RT-PCR in this study, Table S3: Expression of candidate genes regulating trichome cell differentiation, Table S4: Ka/Ks estimated for homolog genes between two poplar species, Table S5: Functional divergence estimated for the two poplar species, Table S6: Summary of Illumina sequencing data and their map ratios, Table S7: Cis-acting regulatory elements in the promoter regions of candidate genes, Table S8: DEGs annotated on the basis of *Arabidopsis* annotations.
