# Supplementary material for: Genome-Wide Survey Reveals Transcriptional Differences Underlying the Contrasting Trichome Phenotypes of Two Sister Desert Poplars
Source: Genes (Basel). 2016 Dec 1;7(12):111. doi: 10.3390/genes7120111 (PMC5192487; doi:10.3390/genes7120111)
Supplement: Supplementary file 1 [file genes-07-00111-s001.zip › genes-144496-supplementary-final/genes-144496-Supplementary Lists.docx]

**Supplementary files:**

Figure S1: Go annotation of DEGs and whole genome genes by WEGO.

Table S1: Sequences of *P. pruinosa*.

Table S2: Primers used for real-time quantitative RT-PCR in this study.

Table S3: Expression of candidate genes regulating trichome cell differentiation.

Table S4: Ka/Ks estimated for homolog genes between two poplar species.

Table S5: Functional divergence of trichome related genes estimated for two poplar species.

Table S6: Summary of the Illumina sequencing data and their map ratios.

Table S7: Cis-acting regulating elements in the promoter region of candidate genes.

Table S8: DEGs annotated on the basis of *Arabidopsis* annotations.
