# Supplementary material for: Genome-Wide Survey Reveals Transcriptional Differences Underlying the Contrasting Trichome Phenotypes of Two Sister Desert Poplars
Source: Genes (Basel). 2016 Dec 1;7(12):111. doi: 10.3390/genes7120111 (PMC5192487; doi:10.3390/genes7120111)
Supplement: Supplementary file 1 [file genes-07-00111-s001.zip › genes-144496-supplementary-final/Table S6 Summary of the Illumina sequencing data and their map ratios-english.docx]

**Table S6.** Summary of the Illumina sequencing data and their map ratios.

| **Samples** | **Length (bp)** | **Reads Number** | **Total Bases (bp)** | **Map Ratios** |
| --- | --- | --- | --- | --- |
| Peu01 | 126 | 40163682 | 5059293709 | 0.594410592 |
| Peu02 | 126 | 41700274 | 5253143406 | 0.648073655 |
| Peu03 | 126 | 33750512 | 4251856916 | 0.610367363 |
| Ppr01 | 126 | 38614618 | 4864516239 | 0.592999806 |
| Ppr02 | 126 | 35415190 | 4461570301 | 0.599494567 |
| Ppr03 | 126 | 32716288 | 4120978900 | 0.609424914 |
