# Supplementary figures and images for: Genome-Wide Survey Reveals Transcriptional Differences Underlying the Contrasting Trichome Phenotypes of Two Sister Desert Poplars
Source: Genes (Basel). 2016 Dec 1;7(12):111. doi: 10.3390/genes7120111 (PMC5192487; doi:10.3390/genes7120111)

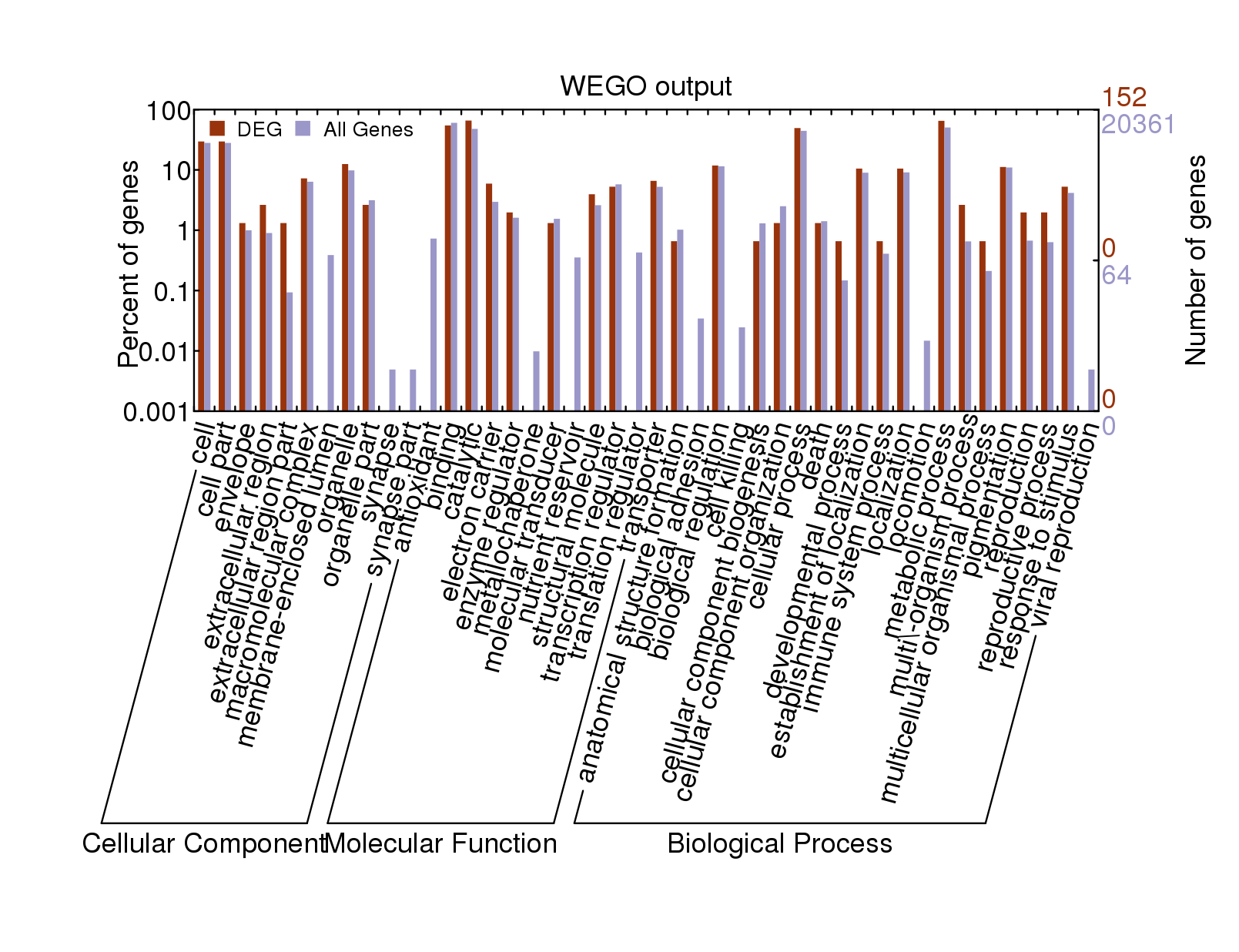


**Figure S1.** Go annotation of DEGs and whole genome genes by WEGO.

Supplement: Supplementary file 1 [file genes-07-00111-s001.zip › genes-144496-supplementary-final/genes-144496-Supplementary-Figure S1 Go annotation of DEGs and whole genome genes by WEGO.docx]
